# Hypersonic acoustic wave control via stealthy hyperuniform phononic nanostructures

**DOI:** 10.1126/sciadv.adw7205

**Published:** 2025-08-06

**Authors:** Michele Diego, Jade Hardouin, Gabrielle Mazevet-Schargrod, Matteo Pirro, Byunggi Kim, Roman Anufriev, Masahiro Nomura

**Affiliations:** ^1^ Institute of Industrial Science, The University of Tokyo, Tokyo 153-8505, Japan.; ^2^ École Polytechnique, Palaiseau 91120, France.; ^3^ ESIEE Paris Cité Descartes, Noisy-le-Grand 93160, France.; ^4^ Department of Mechanical Engineering, School of Engineering, Institute of Science Tokyo, Tokyo 152-8550, Japan.; ^5^ Laboratory for Integrated Micro and Mechatronic Systems, CNRS-IIS IRL 2820, The University of Tokyo, Tokyo 153-8505, Japan.

## Abstract

Controlling hypersonic surface acoustic waves is crucial for advanced phononic devices such as high-frequency filters, sensors, and quantum computing components. While periodic phononic crystals enable precise bandgap engineering, their ability to suppress acoustic waves is limited to specific frequency ranges. Here, we experimentally demonstrate surface acoustic wave control using a hyperuniform arrangement of gold nanopillars on a lithium niobate layer. The hyperuniform structure, exhibiting characteristics of both random and ordered systems, leads to broad-range acoustic transmission reduction and bandgap-like regions of particularly strong suppression. By integrating linear and S-shaped waveguides into the hyperuniform pattern, we achieve efficient waveguiding at frequencies within these bandgaps. Both simulations and experiments confirm high transmission through the waveguides, demonstrating the flexibility of hyperuniform structures to support complex waveguide shapes. These findings provide an alternative approach to overcome limitations of traditional phononic crystals and advance acoustic technologies such as mechanical quantum computing and smartphone filters.

## Teaser

Hyperuniformity-distributed nanopillars manipulate acoustic wave propagation and support complex waveguide designs.

## INTRODUCTION

The manipulation of hypersonic acoustic waves is fundamental for applications in sensors (*1*, *2*), optomechanics (*3*–*5*), topological phononics (*6*–*8*), and mechanical quantum computing (*9*–*11*). For example, the latter requires thermal insulation at sub-kelvin temperatures, which involves suppression of acoustic waves at gigahertz frequencies. Now, well-established systems for nanoscale acoustic wave manipulation are phononic crystals (*12*–*15*), structures consisting of an ordered periodic array of artificially fabricated scatterers. At the nanoscale, these scatterers are typically implemented as holes in thin membranes (*16*, *17*) or pillars on the surface of substrates (*18*, *19*). However, phononic crystals typically target a specific frequency window rather than a wide frequency range (*20*). Moreover, this target window becomes even narrower due to slight imperfections in their practical implementation (*21*). Thus, resistance to perturbations remains both a fundamental question and a practical bottleneck in phononics (*22*–*25*).

In this context, hyperuniform structures are a unique class of materials formed by scatterers arranged with a distribution that falls between order and disorder (*26*). While these structures are nonperiodic and disordered, they retain properties typically associated with crystals, such as short-range local correlations and long-range uniformity. This behavior is typically described by the structure factor *S*(*k*), which reflects density correlations in the reciprocal *k*-space. In general, periodic lattices exhibit sharp peaks in *S*(*k*), systems with suppressed long-range density fluctuations show S(k)→0 as k→0 , while uncorrelated disordered media display a noisy *S*(*k*) behavior around one. Disordered hyperuniform systems can exhibit all of these characteristics: *S*(*k*) approaches zero as the **k**-vector tends to zero, indicating suppressed density fluctuations, and increases to unity for large *k*. Additionally, these systems can show broad peaks at *k*-values corresponding to the average interscatterer distances (*27*).

One particularly interesting class of hyperuniform structures requires S(k)=0 below a certain k=K threshold (*28*). This class is called “stealthy” hyperuniform, and it is widely used in optics due to large and robust photonic bandgaps (*29*–*34*). However, in phononics, hyperuniform structures are yet to be explored. To the best of our knowledge, phononic hyperuniform structures have been studied either theoretically (*35*) or at sub-megahertz frequency range (*36*).

In this study, we investigate a hyperuniform acoustic structure composed of nanoscale gold pillars arranged according to a disordered stealthy hyperuniform distribution on a lithium niobate layer. Interdigital transducers (IDTs) are used to excite and detect surface acoustic waves, exploiting the piezoelectric properties of lithium niobate. This technique enables the measurement of hypersonic transmission spectra in the gigahertz range. By comparing wave transmission with and without the gold hyperuniform structure, we show its impact on acoustic wave propagation. Fundamentally, we aim to demonstrate that hyperuniform structures hinder acoustic waves across a broad frequency range, with bandgap-like regions that offer waveguiding capability due to strong suppression, thus offering an alternative to phononic crystals.

## RESULTS

### Hyperuniform pillars nanostructure

**Fig. 1.**
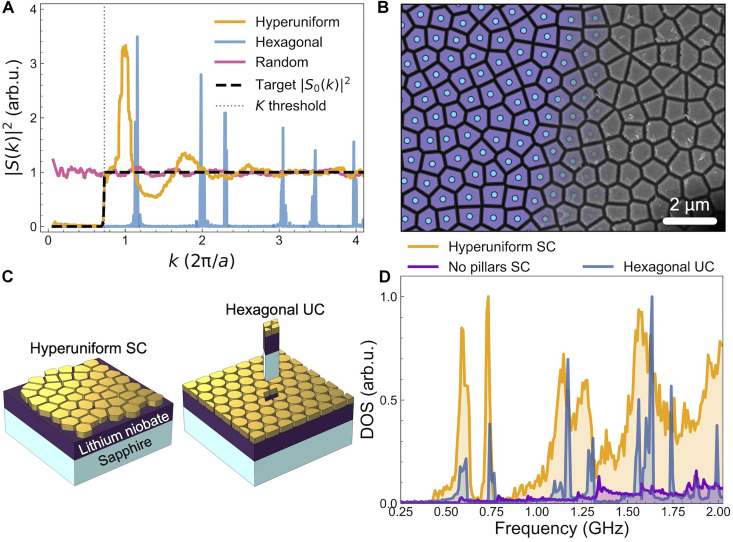
Gold hyperuniform nanostructure on lithium niobate. (**A**) Radially integrated structure factor for the optimized hyperuniform distribution and the target structure factor used as input for the algorithm to generate the hyperuniform pattern. For comparison, the structure factors of a hexagonal lattice (with lattice constant equal to *a*, the average point spacing in the hyperuniform structure) and a random distribution of points are also shown. (**B**) Portion of the hyperuniform distribution (blue dots), its Voronoi cells (purple areas) separated by black gaps (left). The pattern gradually transitions into the scanning electron microscope image of the fabricated structure (right), illustrating the connection between the theoretical design and the experimental realization. The structure is formed by gold pillars of 330-nm height deposited according to the hyperuniform pattern on 1-μm-thick lithium niobate layer upon sapphire. (**C**) Sketch of a portion of the hyperuniform structure, used as an approximate supercell (SC), alongside a portion of a close-packed hexagonal phononic crystal with pillars of the same height and minimum gap as those in the hyperuniform structure. The unit cell (UC) of the phononic crystal is extracted for better visualization. (**D**) DOS of the supercell with and without pillars, compared with the density of state (DOS) of the hexagonal phononic crystal. arb.u., arbitrary units.

The hyperuniform structure consists of gold pillars of 330-nm height deposited on a 1-μm-thick lithium niobate layer atop a sapphire substrate. The pillars are arranged following a stealthy hyperuniform distribution, obtained using an optimization protocol similar to those previously reported in the literature (*37*). Initially, we provided the optimization algorithm with a disordered distribution of points generated by perturbing a hexagonal lattice. The algorithm computes the structure factor *S*(*k*) and evaluates its deviation from a target structure factor $S_{0}\left(k\right)$ designed to enforce a sharp transition from zero to one at k=K . It then iteratively adjusts the point distribution to minimize this deviation. The value of *K* was selected to ensure a high degree of stealthiness in the structure (*38*) (see the “Hyperuniform pattern” section). Figure 1A shows the radially integrated structure factor of the final hyperuniform distribution, together with those of a close-packed hexagonal lattice and a completely random distribution for comparison. The hyperuniform structure exhibits a *S*(*k*) equal to zero for k<K , which then sharply rises to one, following the target structure factor behavior. The broad peaks in the hyperuniform *S*(*k*) derive from short-range correlations between neighboring points, recalling the peaks found for periodic lattices.

To assign physical dimensions to the points in the final distribution, we conducted a Voronoi tessellation (*28*). This involves partitioning the space into regions, or Voronoi “cells,” where each cell is centered around a specific point of the hyperuniform distribution and encloses the area that is closer to that point than to any other. Last, to separate the different cells, we introduced gaps of uniform width between them, ensuring a consistent distance between the cell borders (see the “Hyperuniform pattern” section).

Figure 1B shows on the left a portion of the final hyperuniform point distribution and Voronoi cells, separated by gaps. The image transitions to semitransparency toward the right, gradually unveiling a scanning electron microscopy image of the actual experimental sample extending to the edge. The sample is obtained by drawing the Voronoi cells pattern using electron beam lithography, followed by the deposition of gold. Last, a liftoff process is performed to remove excess gold, revealing the hyperuniform structure. The Voronoi tessellation ensures a high surface coverage, which contributes to a higher interaction with the underlying layer (*39*). The combination of hyperuniform distribution and Voronoi tessellation introduces geometric variability among the pillars. These have a center-to-center distance of 800 to 900 nm, with gap widths ranging from 80 to 95 nm.

To understand the implications of this size dispersion, we compare the density of states (DOS) of the hyperuniform structure with that of a hexagonal phononic crystal composed of identical cylindrical pillars. These pillars have the same height as those in the hyperuniform structure, and the gap at the minimum distance between them is comparable (90 nm).

To calculate the DOS for the hyperuniform structure, we approximate the system using a sufficiently large supercell that encompasses many pillars, effectively accounting for the nonperiodic arrangement and shape variations that preclude the definition of a specific unit cell. Figure 1C shows a sketch of such a supercell, alongside a portion of the hexagonal phononic crystal where the actual unit cell is extracted from the periodic structure. Figure 1D displays the normalized DOS for these systems, as well as that for the large supercell but without the pillars, normalized by the one with the pillars. The presence of the pillars significantly increases the DOS, thereby altering the acoustic properties of the lithium niobate layer. However, a clear difference emerges between the DOS of the hyperuniform structure and that of the hexagonal phononic crystal. In the hexagonal case, the identical pillars with the same resonant frequencies lead to narrow peaks, while, in the hyperuniform structure, variations in pillar shapes cause shifts in their resonance frequencies, resulting in broader peaks and a wider dispersion in the DOS. As we will see, this has a substantial impact on the propagation of surface acoustic waves. In the hyperuniform structure, surface acoustic waves can interact with the pillars over a larger frequency range.

### Effect on acoustic waves transmission

**Fig. 2.**
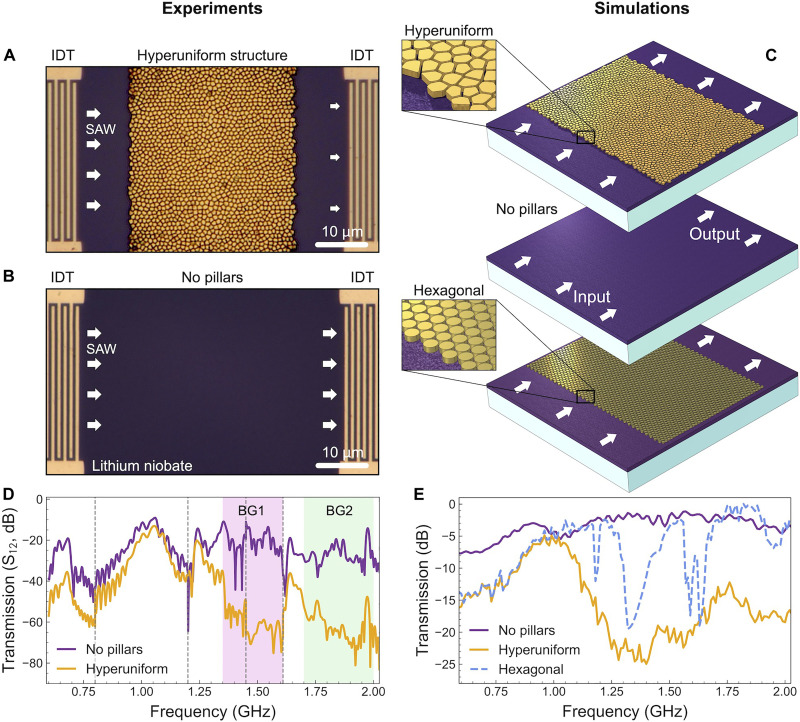
Effect of the hyperuniform structure on the transmission of acoustic waves. (**A** and **B**) Optical microscope images of lithium niobate samples, with (A) and without (B) the gold pillars forming the hyperuniform structure. IDTs at the edges of the structures are used to generate and detect surface acoustic waves (SAW). (**C**) Simulation models of the same structures, used to compare experimental and theoretical transmission spectra, along with the hexagonal phononic crystal for direct theoretical comparison with the hyperuniform structure. (**D**) Experimentally measured transmission spectra in lithium niobate with and without the gold hyperuniform structure. Vertical dashed lines separate measurements with different IDTs, and the experimentally observed bandgap-like regions, BG1 and BG, are highlighted for clarity. (**E**) Simulated transmission spectra in lithium niobate with and without the gold hyperuniform structure, as well as for the hexagonal phononic crystal.

To evaluate the influence of the hyperuniform structure on acoustic wave propagation in lithium niobate, we measured and simulated transmission spectra of acoustic waves with and without the presence of the hyperuniform pillar structure. For the experimental measurements, we fabricated chirped IDTs designed to excite and detect acoustic waves. Figure 2 shows microscope images of the system with IDTs on the sides, both with (A) and without (B) the hyperuniform structure.

Simulations were conducted using the finite element method.  Figure 2C presents the simulation models for the systems with and without the hyperuniform structure, together with the previously discussed hexagonal phononic crystal, enabling a direct comparison of the performance of the two pillar-based configurations. In these simulations, an acoustic excitation with a modulated frequency is applied at the left boundary of the system. For each frequency, the transmission is calculated as the ratio of the elastic energy output on the right side to the input on the left side. This approach mimics the experiment, where one IDT generates the acoustic wave and the other detects the transmission (for more details, refer to the Materials and Methods).

Figure 2 (D and E) shows the transmission results respectively obtained from the experiments and simulations. Although a direct quantitative comparison between the measured and calculated spectra is not possible due to various loss mechanisms in the real sample, the two approaches are generally consistent. For the system without pillars, the transmission shows a slight gradual increase up to 1.1 GHz, eventually reaching a consistent value.

The measurements are obtained using five distinct IDTs, each designed to excite a specific frequency range. Measurements from these IDTs are gated and combined, with each dataset cut and joined to the next. Vertical dashed lines in Fig. 2D indicate the transition points between measurements from different IDTs. The criterion for determining these transition points is to select, for each frequency range, the IDT that exhibits the strongest excitation for the system without pillars. Dips in the transmission spectrum, such as those observed around 0.8 or 1.2 GHz, are attributed to weak excitation by the IDTs rather than being intrinsic properties of the system. Crucially, the 1.25- to 2-GHz range, in which the key physics of this study occurs, is excited evenly.

In the presence of the hyperuniform structure, certain features, such as the experimental peak near 1.65 GHz, deviate from the simulated results, likely due to imperfections in pillar fabrication and adhesion to the substrate. Nevertheless, both methods agree on the main features, revealing a substantial modification in transmission due to the hyperuniform pillars arrangement and highlighting its effectiveness in engineering acoustic wave propagation.

In the lower part of the spectrum, around 0.65 GHz, both the measured and calculated transmissions are reduced compared to the system without pillars. Around 1 GHz, the hyperuniform transmission reaches the no-pillar one, driven by a pronounced peak in the hyperuniform structure response. Beyond 1.35 GHz, both experiments and simulations reveal that the hyperuniform structure induces a broad and substantial decrease in transmission.

The broadness of this frequency window is particularly notable when compared to the simulated transmission shown in Fig. 2E for the hexagonal phononic crystal. Unlike the hyperuniform structure, the hexagonal crystal transmission exhibits sharp dips at specific frequencies. These dips coincide with peaks in the DOS of the hexagonal crystal (Fig. 1D), indicating that the transmission suppression is driven by interactions between the acoustic wave and the local resonances introduced by the pillars, as reported for other pillar-based phononic crystals (*40*). Thus, for the hyperuniform structure, we attribute the broad range of acoustic wave suppression to the broad peaks in the DOS, which result from the variation in pillar shapes and dimensions.

Within this broad frequency window, two distinct regions exhibit particularly low transmission values. Experimental measurements reveal near-total transmission suppression in the ranges of 1.35 to 1.6 GHz and 1.7 to 2 GHz. Due to the substantial transmission suppression, even if not associated with a complete absence of modes in the DOS, we refer to these ranges as effective bandgap-like regions (*41*). These regions are labeled as BG1 and BG2.

### Waveguides

**Fig. 3.**
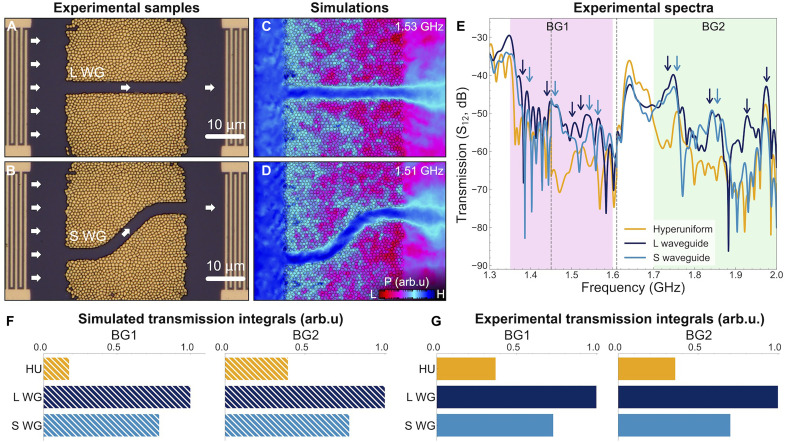
Transmission through waveguides. (**A** and **B**) Optical microscope images of hyperuniform structure with a linear waveguide (L WG) and an S-shaped waveguide (S WG). (**C** and **D**) Simulated acoustic Poynting vector (*P*) at frequencies within BG1, in the presence of hyperuniform structures with two waveguide shapes. The excitation is provided at the left of the structure, as in Fig. 2C. The colormaps are displayed on a logarithmic scale, with the blue color within the waveguides indicating that they support modes with higher transmission. (**E**) Transmission measurements ( $S_{12}$ ) comparison between the hyperuniform structure without waveguide and with the two waveguides. Arrows indicate peaks at frequencies of high transmission, associated with modes enabled by the waveguides. (**F** and **G**) Histograms of the integrated transmission in simulations (F) and experimental measurements (G) within the effective bandgaps for the hyperuniform structure without and with the waveguides.

The emergence of effective bandgaps associated with the hyperuniform structure suggests the potential for designing acoustic waveguides capable of transmitting waves at frequencies within these bandgaps. Figure 3A shows a microscope image of the hyperuniform structure with a linear waveguide created by removing pillars along a 3-μm-width strip. Additionally, we designed an S-shaped waveguide, shown in Fig. 3B, to test a freeform waveguide shape. As shown in optical experiments (*42*), hyperuniform patterns enable the design of freeform waveguides that are not achievable with conventional photonic/phononic crystals.

Using finite element method simulations, we calculated the propagation of acoustic waves in these waveguides, following the same approach as in previous transmission simulations. Figure 3 (C and D) presents colormaps of the acoustic Poynting vector for the two systems when excited at frequencies within BG1. The Poynting vector is substantially higher within the waveguides, illustrating their effectiveness in transmitting acoustic waves.

To observe the wave guidance experimentally, we measured the transmission spectra for the systems with waveguides via IDTs. Figure 3E compares these transmission spectra with that of the hyperuniform structure without waveguides. Outside the bandgap-like regions, the transmission is similar across all structures, with shared peaks observed at ~1.35 and 1.65 GHz. Within the bandgaps, the transmission is higher in the presence of waveguides, exhibiting distinct peaks marked by arrows. These peaks are interpreted as modes supported by the waveguides.

This result is further demonstrated through the integration of the transmission spectra over the bandgap-like ranges. Figure 3 (F and G) presents the histograms of the transmission (normalized to the highest peak) obtained from simulations and experiments, respectively. For each bandgap, we compare the transmission of the hyperuniform structure and the systems with the two waveguides.

Overall, both simulations and experimental measurements agree that waveguides allow a higher transmission of acoustic waves. Among all configurations, the linear waveguide consistently achieves the highest transmission. Nevertheless, the S-shaped waveguide also achieves high transmission values within both effective bandgaps. Note that, in both the experiment and simulations, the transmission is averaged across the entire hyperuniform length, because, experimentally, it is not possible to probe acoustic waves only at the waveguide exit.

These results demonstrate that the hyperuniform structure enables the creation of phononic waveguides, including those with freeform shapes like the S-shaped design.

## DISCUSSION

This work experimentally demonstrates a practical application of hyperuniformity in nanophononics. Our results illustrate the control of hypersonic surface acoustic waves using a hyperuniform distribution of gold pillars on a substrate.

We first investigated the impact of the hyperuniform pattern on acoustic wave transmission. Our findings reveal that the transmission is significantly reduced by the presence of gold pillars, with experimental measurements and simulations showing overall consistency. Notably, the hyperuniform structure hinders the propagation of acoustic waves over a broad gigahertz range, including bandgap-like regions of particularly strong suppression.

We have seen that the large suppression window emerges from the broad peaks in the DOS of the hyperuniform structure. These are associated with the variations in pillar size and geometry introduced by the hyperuniform distribution and Voronoi tessellation, in contrast to traditional phononic crystals composed of identical pillars. We emphasize that the tessellation process plays a critical role, as assigning identical pillars to each point in the hyperuniform distribution would prevent the DOS from exhibiting peak broadening, thereby eliminating the mechanism for broad suppression demonstrated in this work. The same argument applies to identical pillars arranged in a random distribution, with the additional drawback that the lack of spatial correlation would result in some points being arbitrarily close, causing overlapping of the pillars.

We then exploited the effective bandgaps to design linear and S-shaped waveguides by removing pillars from the hyperuniform structure. Both simulations and experiments confirm that these waveguides allow transmission within the bandgaps. Thus, the hyperuniform structure supports freeform waveguides, such as the S-shaped configuration. This feature offers design flexibility, enabling alternative device architectures with improved functionality as compared to traditional periodic phononic crystals.

## MATERIALS AND METHODS

### Hyperuniform pattern

The hyperuniform distribution was generated using *N* = 418 points. The threshold **k**-vector was chosen to be $K=\sqrt{8\pi N}$ , a choice that ensures a high level of stealthiness (*38*). This is quantified by the parameter χ = 0.5, which reflects the degree of correlation in the system (*43*).

To optimize the distribution, we minimized the function

$$F=\sum_{k}{|S\left(R_{N},k\right)-S_{0}\left(k\right)|}^{2}$$ (1)

where

$$S\left(R_{N},k\right)=\frac{1}{N}\sum_{i,j=1}^{N}e^{i\left(r_{i}-r_{j}\right)k}$$ (2)

is the structure factor for the specific distribution of points defined by the positions $R_{N}=\left\{\;r_{1},r_{2},\ldots ,r_{N}\;\right\}$ and the target structure factor is given by

$$S_{0}\left(k\right)=\left\{\;\begin{array}{cc} \;K^{-\alpha }k^{\alpha } & \text{for}\;0<k<K\\ \;1 & \text{for}\;k\geq K \end{array}\right.$$ (3)

where we set α = 100, ensuring that $S_{0}\left(k\right)$ undergoes a sharp transition from near zero to one at *K*. Note that, in the Results, we always use the scalar notation for the wave vector ( k=∣k∣ ) instead of the vectorial notation. This choice is made for simplicity, as only the magnitude is relevant due to the isotropic nature of the hyperuniform structure. The minimization process was carried out in Python environment using the conjugate gradient method via the scipy.optimize.minimize function. The convergence criterion was defined by the gradient tolerance, set to $10^{-4}$ , meaning the optimization was considered converged when the norm of the gradient of the objective function fell below this threshold.

**Fig. 4.**
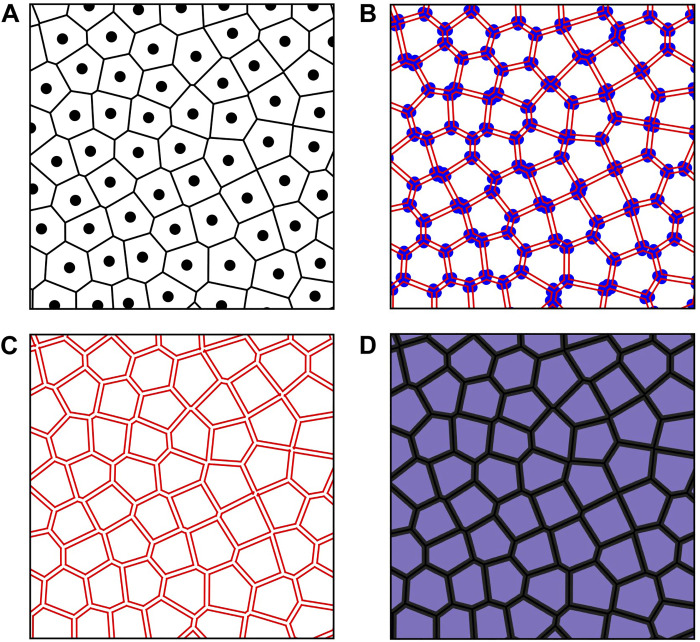
Steps for the separation of the Voronoi cells. (**A**) Point distribution with Voronoi tessellation. (**B**) Identification of the vertex. (**C**) Creation of the borders with equal width. (**D**) Final separated Voronoi cells.

After obtaining the hyperuniform distribution, the Voronoi tessellation was obtained again in Python environment using the scipy.spatial.Voronoi method. To create separated cells, we assigned a specified width to the borders of the Voronoi cells. This process involved extensive geometric manipulation using vertex coordinates and edge lists. Figure 4 illustrates some of the steps involved. Ensuring an equal width distance between the cells is crucial for the fabrication feasibility of the structure and for maintaining a uniform average diameter of the resulting cells.

The hyperuniform structure obtained with 418 points yields an experimental sample of ~15 μm by 15 μm for an average pillar distance of 800 to 900 nm. However, to match the scale of the IDTs, the experimental sample dimensions need to be around 30 μm by 45 μm. Calculating a hyperuniform structure with that number of points would be computationally prohibitive. Therefore, to achieve the desired dimensions, we assembled a 2×3 matrix of hyperuniform 418-point squares prior to performing the Voronoi tessellation, ensuring smooth transitions between adjacent regions. Consequently, our experimental samples effectively consist of a 2×3 repetition of a large 15 μm–by–15 μm supercell containing ~418 pillars, with some pillars at the interfaces sfshared between supercells.

### Finite elements simulations

All simulations were performed using the finite element method. The gold pillars and sapphire substrate were modeled as isotropic materials, with densities set to $\rho_{\text{Au}}=19,300\;\text{kg}/m^{3}$ and $\rho_{{\text{Al}}_{2}O_{3}}=3965\;\text{kg}/m^{3}$ , the Young’s moduli to $E_{\text{Au}}=70\;\text{GPa}$ and $E_{{\text{Al}}_{2}O_{3}}=400\;\text{GPa}$ , and the Poisson’s ratios to $\nu_{\text{Au}}=0.44$ and $\nu_{{\text{Al}}_{2}O_{3}}=0.22$ (*44*–*46*). The lithium niobate layer was modeled using a density $\rho_{\text{LN}}=4700\;\text{kg}/m^{3}$ and the full anisotropic elastic tensor, with stiffness constants $C_{11}$ = 203, $C_{12}$ = 53, $C_{13}$ = 75, $C_{14}$ = 9, $C_{33}$ = 243, and $C_{44}$ = 60 GPa (*47*). The tensor was rotated to match the crystallographic orientation of the experimental sample.

**Fig. 5.**
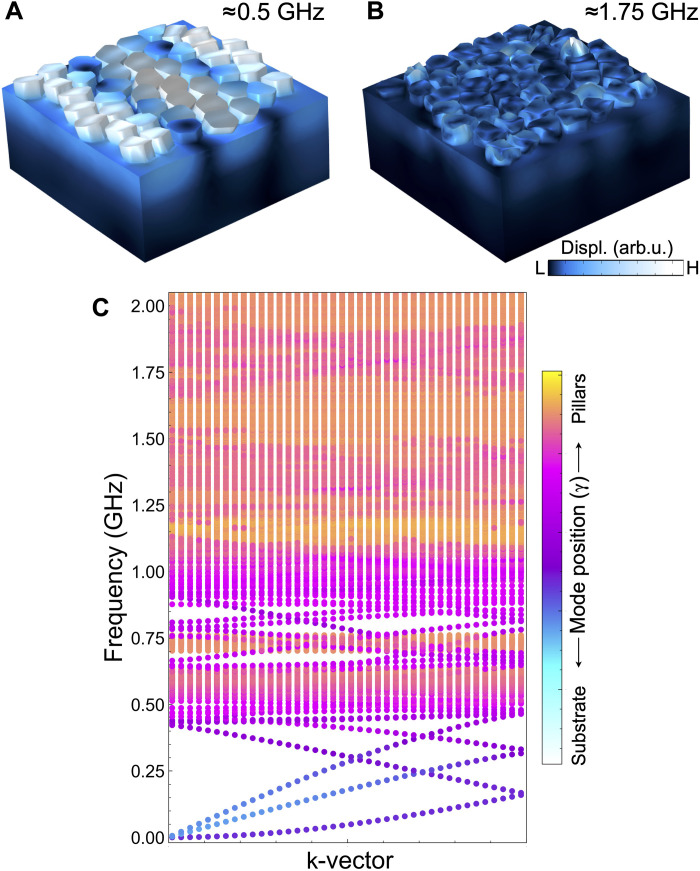
Phonon dispersion of the hyperuniform supercell. (**A** and **B**) Displacement for eigenmodes in the supercell at low frequency (A) and high frequency (B). (**C**) Total phonon dispersion associated with the supercell (Fig. 1C), where each mode is colored according to its vertical position in the cell. L, low; H, high.

Figure 5 shows the phonon dispersion of the supercell depicted in Fig. 1C. To calculate it, we applied Floquet periodic boundary conditions to the lateral sides of the supercell. The sapphire substrate is approximated as 1.5 μm thick with a free boundary condition at the bottom. Although the experimental substrate is much thicker, this choice does not affect the DOS, which is dominated by modes near the pillars. We then computed the eigenfrequencies of the system while sweeping the **k**-vector in from 0 to $\pi /L_{\text{sc}}$ , where $L_{\text{sc}}=6.25$ μm is the length of the supercell side. The phonon dispersion was calculated only along the experimentally probed direction; however, the isotropy of the hyperuniform pillar distribution ensures that the resulting DOS remains direction independent. Each point in the phonon dispersion is colored according to

$$\gamma =\frac{1}{t+h}\frac{\int_{V}ℰ\;y\;dr}{\int_{V}ℰ\;dr}$$ (4)

where *t* is the thickness of the substrate (sapphire + lithium niobate), *h* is the height of the pillars, ℰ is the elastic energy density, *y* is the vertical component of the position vector, and the integrals are performed over the volume *V* of the supercell. In this way, γ indicates at which height the modes are localized and helps determine whether they correspond to pillar or substrate modes. The phonon dispersion is plotted with substrate modes in front of the pillar modes to enhance the visibility of their features. Peaks (dips) in the theoretical transmission in Fig. 2E approximately correspond to frequency ranges where the modes are more confined within the substrate (pillars).

The densities of states shown in Fig. 1D are calculated from the phonon dispersion by grouping the modes on the basis of their frequency, regardless of their **k**-vector. For the transmission simulations in Figs. 2 and 3, the sapphire substrate is modeled as a 5-μm layer atop a perfectly matched layer, which prevents artificial reflections, ensuring realistic behavior. Similarly, low-reflective boundary conditions are applied at the lateral sides of the system, again to avoid artificial reflections. The input acoustic waves are excited via a mechanical source on a surface on the left of the system, as explicitly modeling the IDTs would be too computationally demanding. However, the input excitation is designed to mimic the effect of realistic IDTs. To replicate the surface excitation generated by IDTs, the excitation boundary is embedded within the lithium niobate layer with a depth of 250 nm. Moreover, the excitation has components along all three axes, a choice justified by simulations of the system without pillars that explicitly include the piezoelectric interaction between the IDTs and lithium niobate, as shown at the end of this section. Also, the transmission simulations do not account for the piezoelectric coupling between lithium niobate and the gold pillars, as dedicated simulations incorporating this effect (see the Supplementary Materials) showed no substantial differences and require a prohibitive computational cost. On the opposite side of the system, an identical boundary is placed to capture the output acoustic waves, simulating the receiver IDT. The transmission is then computed as the ratio of the integrated elastic energies at the output and input boundaries.

**Fig. 6.**
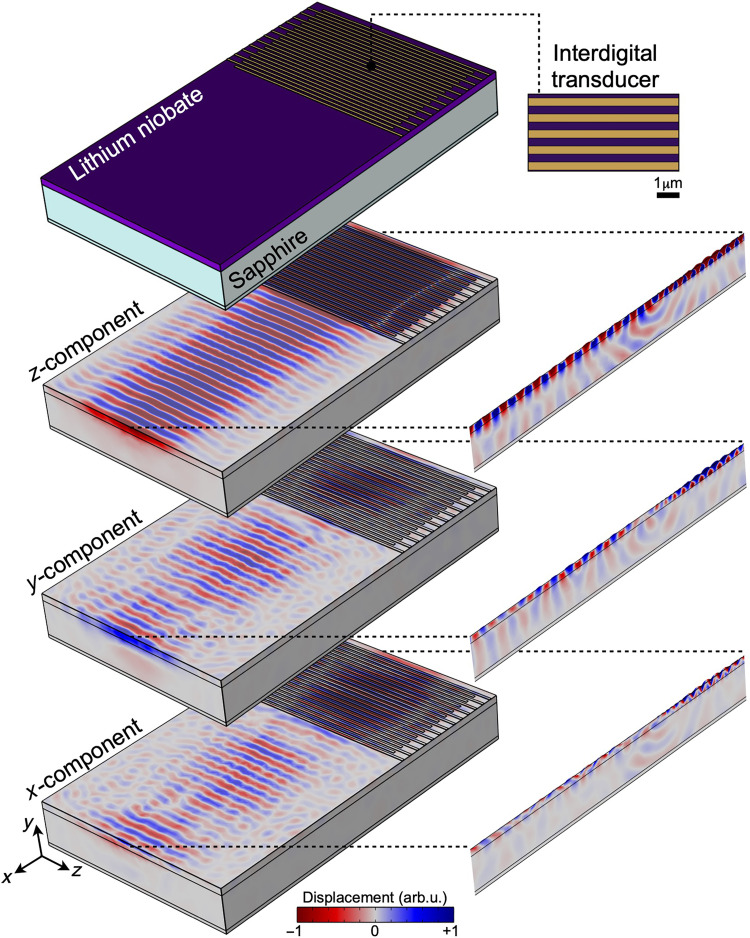
Simulation of acoustic wave excitation by IDTs. The resulting displacement components (*x*, *y*, and *z*) at ~2 GHz are shown in both a three-dimensional view and cross-sectional slices at the center of the system.

Figure 6 illustrates simulations modeling the excitation from the IDTs via the piezoelectric effect. A modulated periodic voltage applied to the electrodes generates a surface acoustic wave at ~2 GHz in the lithium niobate layer. The resulting displacement components (*x*, *y*, and *z*) are visualized in both three-dimensional and cross-sectional views at the center of the system. These results confirm that the excited wave exhibits substantial displacement along all three axes, supporting the chosen design of the mechanical excitation in the transmission simulations.

### Fabrication of the samples

**Fig. 7.**
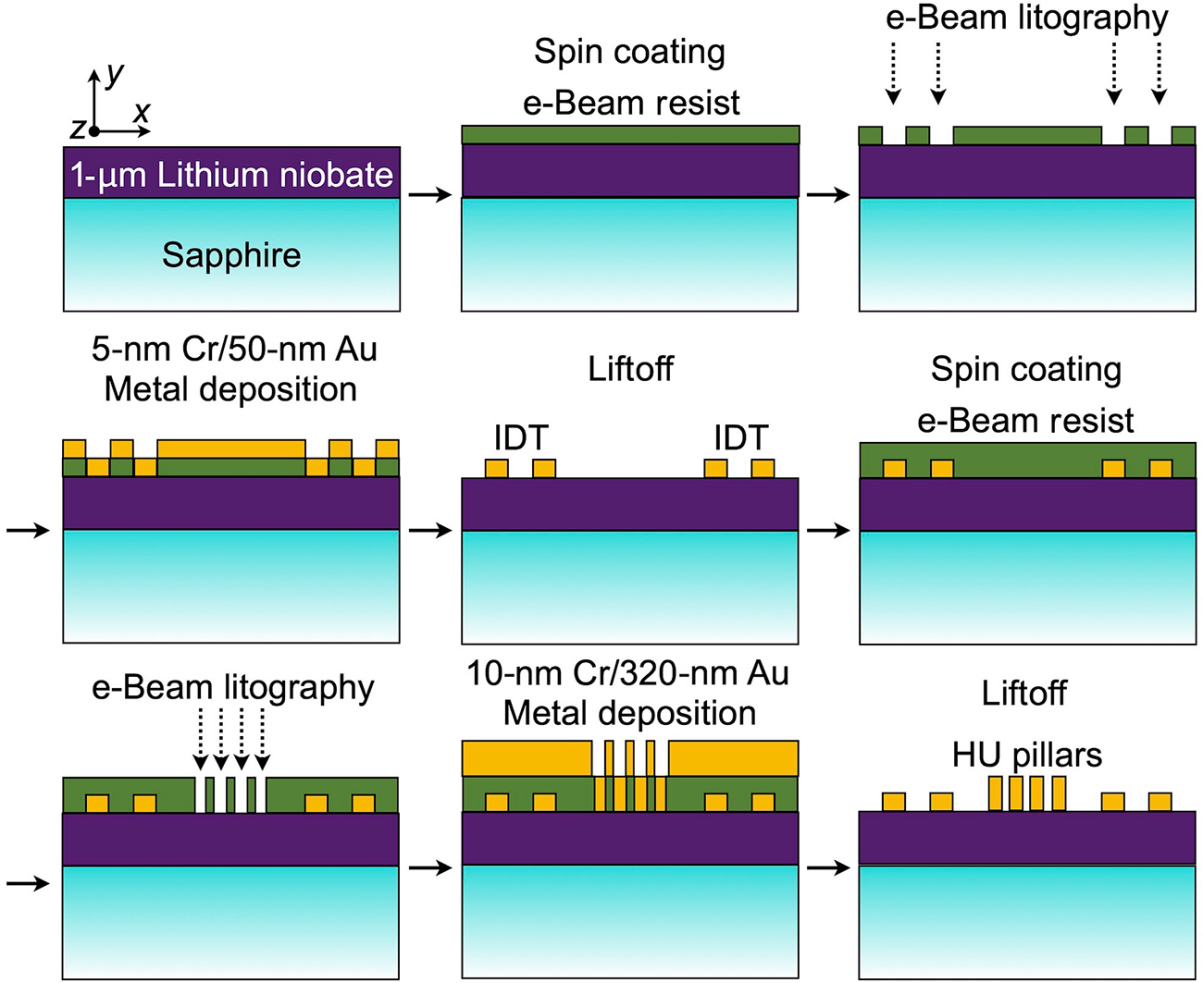
Schematics of the fabrication steps. e-Beam, electron beam; HU, hyperuniform.

The wafer used in this work consists of a commercially available 1-μm-thick layer of *y*-cut lithium niobate on a sapphire substrate, supplied by NGK Insulators Ltd. The fabrication process involved several steps, summarized in Fig. 7. First, an electron beam resist layer with a thickness of ~300 nm was spin coated onto the sample. Electron beam lithography was then performed to pattern the IDTs, which are oriented to excite acoustic waves propagating along the *x* axis. Following this, a physical vapor deposition process was used to deposit 5 nm of chromium and 50 nm of gold. A liftoff process was then carried out to obtain the final form of the IDTs.

Five different IDT types were fabricated, each designed to excite distinct frequency ranges. Each IDT consists of 30 fingers on the ground side and 30 fingers on the signal side. The IDTs are chirped, meaning their finger widths were designed to target a central frequency $f_{0}$ , with the widths gradually varying to excite a frequency range of ~$\pm 15\%\;f_{0}$ . The distance between the hyperuniform structure and the nearest finger of the IDTs is ~10μm.

The same sequence of steps was repeated for fabricating the hyperuniform pillars, with slight modifications. In this case, the resist thickness was increased to ~550 nm, and the deposition involved 10 nm of chromium followed by 320 nm of gold. In the result section, we approximated the pillars as 330 nm of gold, omitting the thin chromium layer whose only role is the adhesion of gold on the substrate.

### IDT measurements

Measurements were performed at room temperature using a vector network analyzer in conjunction with a calibrated microwave probe station. The parameter $S_{12}$ (or $S_{21}$ ) describes the transmission coefficient of the device under test, quantifying the amount of signal injected into one port that is transmitted to the other port. Conversely, $S_{11}$ (or $S_{22}$ ) represents the reflection coefficient at each port, indicating how much of the signal is reflected back to the same port.

**Fig. 8.**
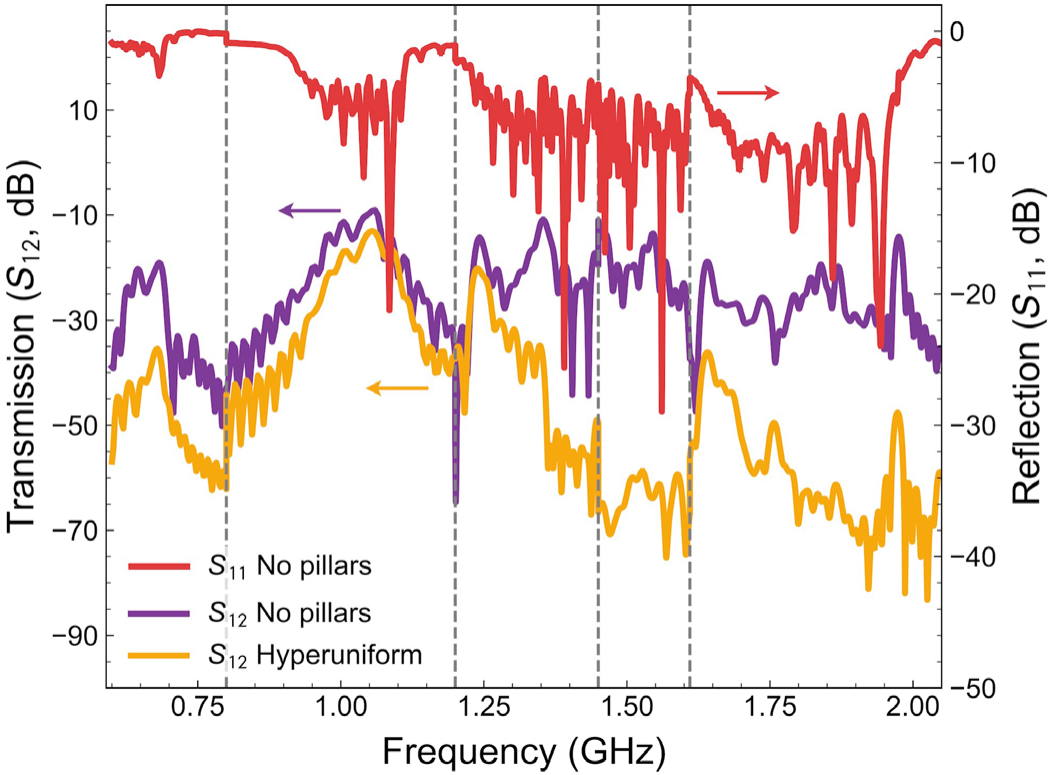
IDT measurements. The transmission (*S*_12_, left axis) in the presence of the hyperuniform structure, and both transmission (*S*_12_, left axis) and reflection (*S*_11_, right axis) without the hyperuniform structure. The reflection shows which ranges of frequencies are properly excited.

Figure 8 illustrates the $S_{12}$ parameter both with and without the hyperuniform structure, as well as the $S_{11}$ parameter in the absence of the hyperuniform structure. The $S_{11}$ parameter identifies the frequencies excited by each IDT. The measurements are first gated to remove the nonacoustic background (*48*, *49*) and then combined to create a unified, coherent spectrum. The criterion for connecting different measurements is to select, within each frequency range, the data from the IDT that exhibits the highest excitation in the absence of the hyperuniform structure. In the 0.6- to 1.2-GHz range, measurements were performed with a step of 2.5 MHz (400 steps per GHz), while, in the 1.2- to 2.25-GHz range, the step size is reduced to 0.5 MHz (2000 steps per GHz). To calculate the integral of the transmission in the bandgap-like regions in Fig. 3G, we converted the $S_{12}$ data to the transmission in linear scale through $T=10^{S_{12}\left(\text{dB}\right)/10}$.
