# Supplementary material for: Hypersonic acoustic wave control via stealthy hyperuniform phononic nanostructures
Source: Sci Adv. 2025 Aug 6;11(32):eadw7205. doi: 10.1126/sciadv.adw7205 (PMC12327447; doi:10.1126/sciadv.adw7205)
Supplement: Supplementary file 1 — Figs. S1 to S3 [file sciadv.adw7205_sm.pdf]

Science Advances
AAAS

Supplementary Materials for

Hypersonic acoustic wave control via stealthy hyperuniform phononic nanostructures

Michele Diego *et al.*

Corresponding author: Michele Diego, diego@iis.u-tokyo.ac.jp; Masahiro Nomura, nomura@iis.u-tokyo.ac.jp

Sci. Adv. **11**, eadw7205 (2025)
DOI: 10.1126/sciadv.adw7205

This PDF file includes:

Figs. S1 to S3

The supplementary material provides additional figures for a better visualization on the suppression of acoustic waves by the hyperuniform structure in comparison with the hexagonal phononic crystal (Figure S1), the waveguiding effect through the hyperuniform structure (Figure S2), and the simulations that included the full mechanical and electrical interactions between lithium niobate and the pillars (Figure S3).

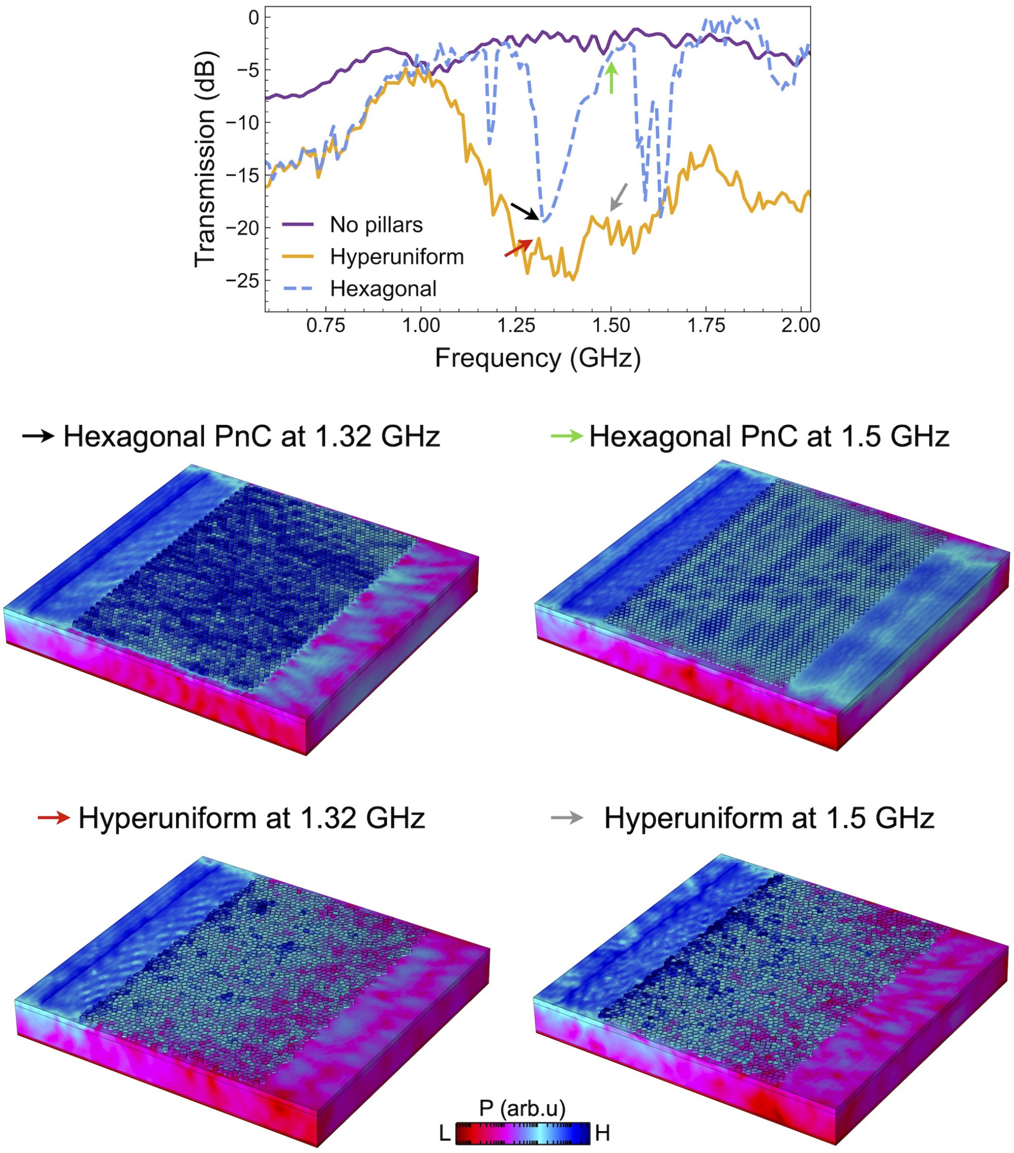

Figure S1. **Simulated transmission of acoustic waves through the hyperuniform structure and the hexagonal phononic crystal.** In addition to the plot presented in the main text, we show 3D colormaps in logarithmic scale of the acoustic Poynting vector for selected frequencies: 1.32 GHz, where both structures exhibit strong suppression, and 1.5 GHz, where strong suppression is observed only for the hyperuniform structure.

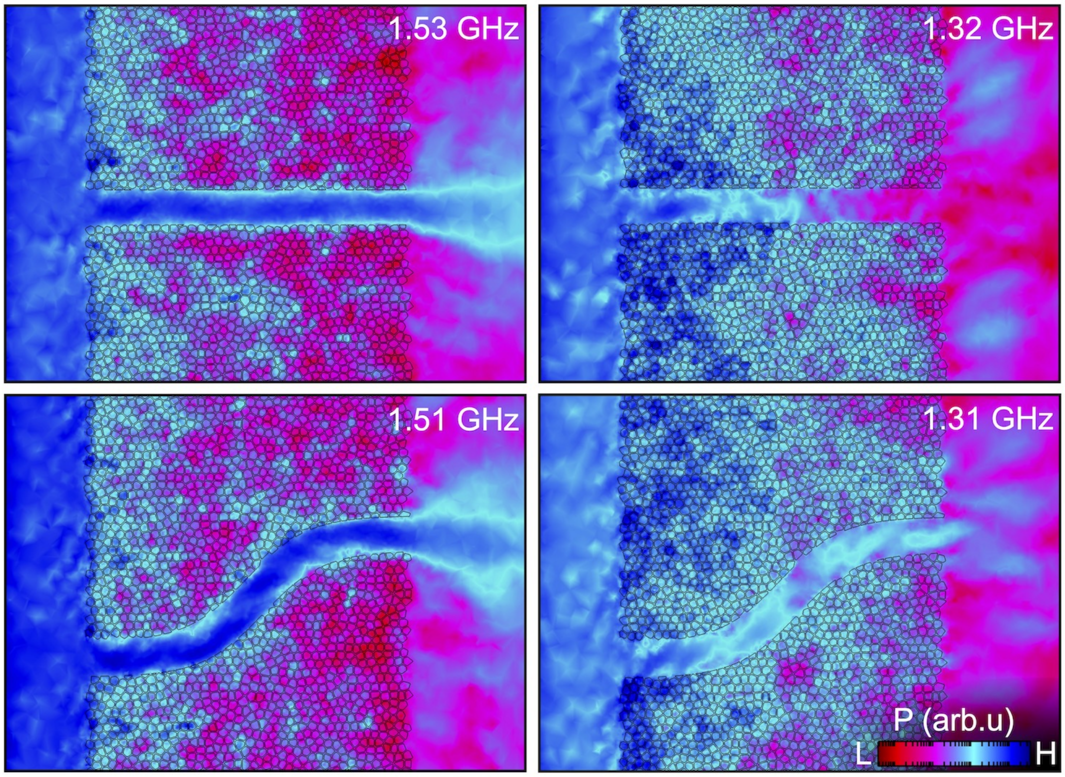

Figure S2. **Simulated transmission of acoustic waves through the hyperuniform structure, featuring linear (top) and S-shaped (bottom) waveguides.** The examples display the acoustic Poynting vector in logarithmic scale at frequencies where waveguiding supports strong transmission (left) and where waveguiding is not observed (right).

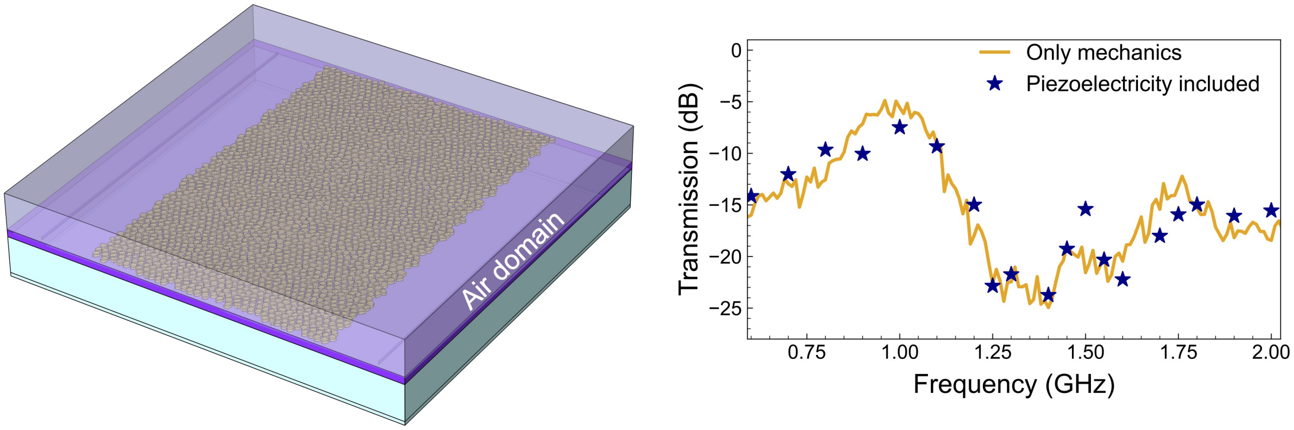

Figure S3. **Simulated transmission of acoustic waves considering the piezoelectric effect and the electric interaction between the lithium niobate layer and the gold pillars.** The model (left) includes an air domain above the structure to account for the electric field around the pillars. Due to the high computational cost, simulations were performed at selected frequencies (blue points in the plot on the right) and compared to the transmission from the main text (yellow curve), where only mechanical interactions are considered. The two approaches agree both qualitatively and quantitatively, with the only exception of one point at around 1.5 GHz. However, even at this frequency, transmission remains strongly suppressed in both models, indicating that the mechanical interaction is the dominant mechanism in describing the system.
